# Temporal trends in the prevalence of major birth defects in China: a nationwide population-based study from 2007 to 2021

**DOI:** 10.1007/s12519-024-00844-9

**Published:** 2024-11-02

**Authors:** Wen-Yan Li, Zhi-Yu Chen, Wen-Li Xu, Yu-Yang Gao, Zhen Liu, Qi Li, Li Dai

**Affiliations:** 1 National Center for Birth Defects Monitoring, West China Second University Hospital, Sichuan University, Chengdu, 610041 Sichuan China; 2 Key Laboratory of Birth Defects and Related Diseases of Women and Children, Ministry of Education, Sichuan University, No. 17 Section 3 Renminnanlu, Chengdu, 610041 China; 3 The Joint Laboratory for Pulmonary Development and Related Diseases, West China Second University Hospital, Sichuan University, Chengdu, 610041 China; 4 NHC Key Laboratory of Chronobiology, Sichuan University, Chengdu, 610041 Sichuan China; 5 Med-X Center for Informatics, Sichuan University, Chengdu, 610041 Sichuan China

**Keywords:** Birth defects, China, Epidemiology, Prevalence, Trend

## Abstract

**Background:**

Birth defects constitute a significant public health issue worldwide, yet there is a lack of comprehensive population-based data for the Chinese population.

**Methods:**

We analyzed data from the China National Population-based Birth Defects Surveillance System from 2007 to 2021, we calculated the prevalence rates of selected birth defects, stratified by maternal residence, geographic region, maternal age, and infant sex. The Joinpoint regression model was utilized to assess trends and annual percent changes in prevalence.

**Results:**

From 2007 to 2021, significant downward trends in prevalence were observed for neural tube defects (NTDs), hydrocephalus, cleft lip with or without palate (CL/P), limb reduction defects (LRD), omphalocele, Down syndrome, and tetralogy of Fallot (TOF). Conversely, upward trends were identified for hypospadias, cleft palate (CP), microtia/anotia, polydactyly, syndactyly, ventricular septal defect (VSD), atrial septal defect/patent foramen ovale (ASD/PFO), and patent ductus arteriosus (PDA). Younger mothers exhibited a higher prevalence of hydrocephalus, gastroschisis, CL/P, and polydactyly, while anotia/microtia, Down syndrome, and congenital heart diseases (CHDs) were more common in mothers aged 35 years or older. Significant variations in the prevalence of anencephalus, spina bifida, CL/P, anorectal atresia/stenosis, hypospadias, polydactyly, syndactyly, VSD, ASD/PFO, and PDA were found across different maternal residences and geographic regions.

**Conclusion:**

This study highlights the diverse trends and prevalence patterns of major birth defects, underscoring the necessity for defect-specific public health interventions.

**Graphical abstract:**

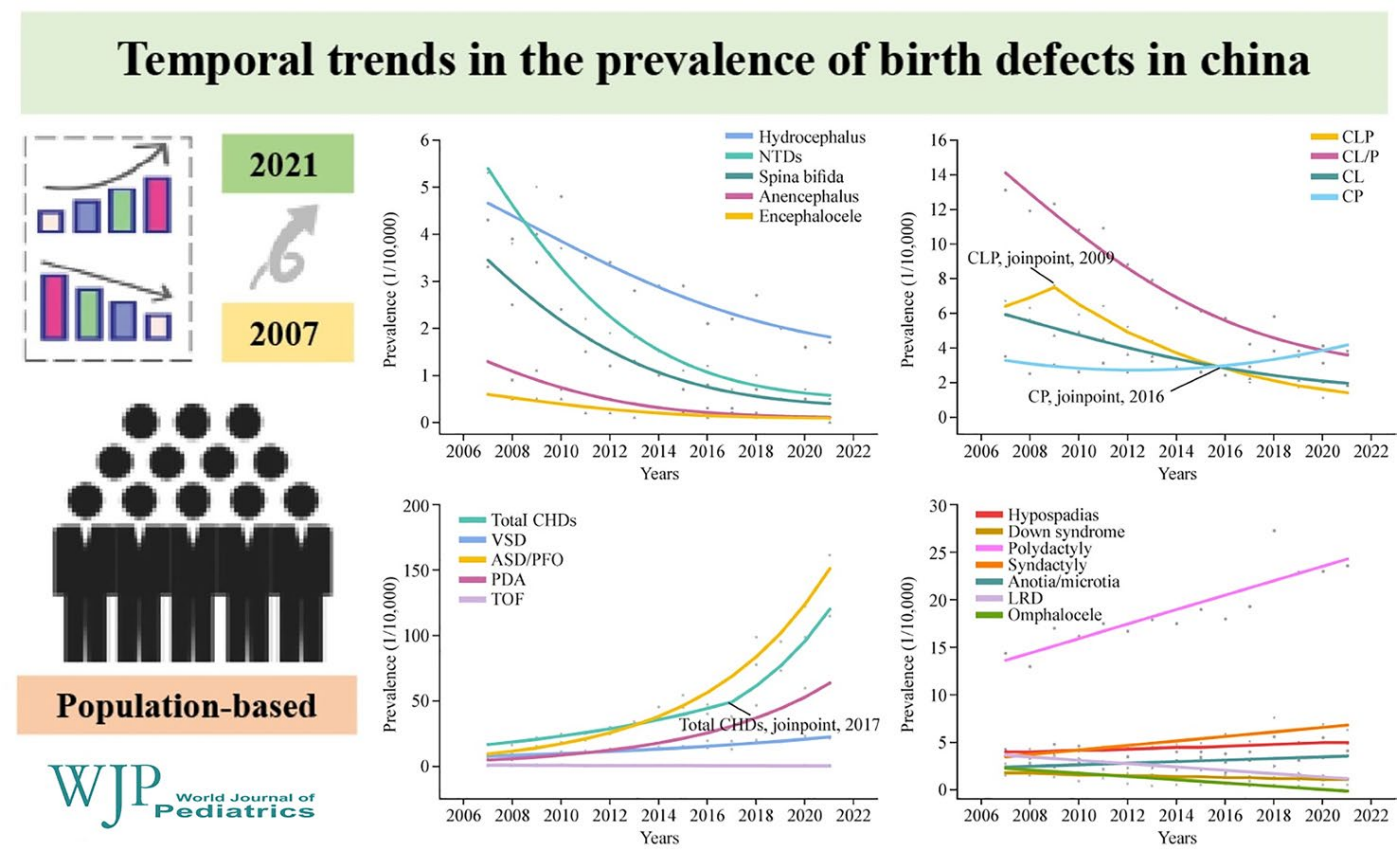

**Supplementary Information:**

The online version contains supplementary material available at 10.1007/s12519-024-00844-9.

## Introduction

Birth defects pose a significant clinical and public health challenge worldwide. Each year, nearly 8 million infants are born with severe birth defects, and tragically, over 3 million of these infants do not survive their fifth birthday [1]. While hospital-based studies offer valuable insights, population-based surveillance is indispensable [2–6]. In China, however, population-based data are scarce. Most studies relied on biased samples from healthcare facilities or provided regional estimates for specific phenotypes or categories of birth defects [7–10].

From 2007 to 2021, China consistently implemented a variety of policies and measures aimed at preventing and controlling birth defects, including folic acid supplementation for women of reproductive age, pregnancy health examinations, and neonatal disease screening. There initiatives have significantly reduced the prevalence of birth defects during this period. Concurrently, China’s fertility policies transitioned from a one-child policy to a universal two-child policy. This shift introduced new challenges for the prevention and control of birth defects, such as increases in advanced maternal age, the use of assisted reproduction techniques (ART), multiparity, and multiple pregnancies. As China’s social economy rapidly developed, there were substantial changes in family income, maternal nutrition, prenatal care, and other socioeconomic and demographic factors. Despite these advancements, reliable, population-based data on the prevalence of major birth defects remains scarce. Understanding the long-term trends in major birth defects is valuable for informing future policy adjustments and interventions.

Utilizing data from the China National Population-based Birth Defects Surveillance System (CNPBDSS) spanning the years 2007 to 2021, our study aimed to comprehensively analyze the prevalence and temporal trends of selected major birth defects within the Chinese population. These findings provide crucial evidence to guide future interventions and inform policy-making efforts.

## Methods

### Study population

Population data from 2007 to 2021 were obtained from the China National Population-based Birth Defects Surveillance System (CNPBDSS), a nationwide system that monitors birth defects across 64 counties or districts in 30 provinces, municipalities, and autonomous regions in China. The CNPBDSS serves as an essential tool for gaining insights into the epidemiology of birth defects throughout the country. To ensure a representative sampling of the local birth population, an urban district and a rural county in each province were selected as surveillance sites. These selections were based on robust maternal and child health infrastructure, high hospital delivery rates exceeding 90%, and systematic maternity management rates also exceeding 90%. These sites provided comprehensive maternal and neonatal care, including screenings for congenial conditions. The study population included fetuses and neonates from 28 weeks of gestation or more, born to women residing in the defined surveillance areas, with follow-up continuing until 42 days post-birth. Any major birth defects diagnosed for the first time during this period were mandatorily reported. All recorded defects were coded by national-level staff in accordance with the International Classification of Diseases, 10th Revision (ICD-10). Further details regarding the data collection procedure and quality control measures can be found in other publications [11, 12].

We conducted a comprehensive analysis of 17 major birth defects frequently assessed through surveillance. These defects encompass a range of conditions, including neural tube defects (NTDs), such as anencephalus (Q00), spina bifida (Q05), and encephalocele (Q01); hydrocephalus (Q03); anotia/microtia (Q16.0 and Q17.2); cleft palate (CP, Q35); cleft lip with or without palate (CL/P, Q36**–**Q37); esophageal atresia/stenosis (Q39.0**–**Q39.3); anorectal atresia/stenosis (Q42); hypospadias (Q54); clubfoot (Q66.0); polydactyly (Q69); syndactyly (Q70); limb reduction defects (LRD, Q71**–**Q73); diaphragmatic hernia (Q79.0); omphalocele (Q79.2); gastroschisis (Q79.3); and Down syndrome (Q90). Additionally, we presented the common minor congenital heart diseases (CHDs), such as atrial septal defect/patent foramen ovale (ASD/PFO, Q21.1) and patent ductus arteriosus (PDA, Q25.0), alongside major ones like ventricular septal defect (VSD, Q21.0), transposition of the great arteries (TGA, Q20.3), and tetralogy of Fallot (TOF, Q21.3). We also present the prevalence rate of total CHDs (Q20**–**Q26), excluding minor cases. The selection of these defects was guided by their clinical significance and alignment with the CNPBDSS, ensuring that our study captures the most common and critical structural birth defects affecting China's perinatal population.

This study was conducted using anonymous surveillance data that did not include identifiable information for the mothers or infants. Therefore, the Medical Ethics Committee of West China Second University Hospital reviewed and approved a waiver of informed consent.

### Statistical analysis

We conducted data cleaning and analysis using R version 4.3.1. The prevalence of birth defects was calculated as the ratio of cases to births per 10,000 births. Prevalence was estimated for individual birth defects and categorized by maternal age (< 20, 20**–**24, 25**–**29, 30**–**34, and ≥ 35 years), infant sex (male or female), maternal residence (urban: cities, urbanized areas, and neighborhood committees; or rural: villages and countryside), and geographic regions (eastern, central, and western, based on geographic location and economic status) [13, 14]. Chi-squared tests were used to examine differences in prevalence between these groups. Joinpoint regression analysis [15] (Joinpoint Regression Program 5.0.2, National Cancer Institute) was utilized to delineate the temporal trends in the prevalence of selected birth defects. This approach allowed us to identify inflection points (joinpoints) that marked significant alterations in trend over the study period. The long-term trends were further segmented based on detected joinpoints. For each segment, we calculated the annual percentage change (APC) along with a 95% confidence interval (CI) to quantify trajectory patterns. As a summary measure of the overall temporal trend from 2007 to 2021, we computed the average annual percentage change (AAPC). The statistical significance of the temporal patterns was evaluated through a Monte Carlo permutation method, with an α level of 0.05.

## Results

From 2007 to 2021, the CNPBDSS recorded a total of 5,091,580 prenatal infants, with 53.0% males and 47.0% females. Notably, 10.4% of these infants were born to mothers aged 35 years or older. Additionally, 46.4% of the infants were from rural areas. The distribution varied geographically, with 47.4% of births in eastern China, 27.2% in central China, and 25.4% in the western region.

Tables 1 and 2 detail the prevalence rates and 95% CIs for each birth defect, stratified by maternal age, infant sex, maternal residence, and geographic region. The most prevalent birth defects identified were CHDs, polydactyly, CL/P, syndactyly, and hypospadias. Certain defects, such as NTDs, CL/P, and gastroschisis, exhibited significantly higher prevalence in rural areas and the western region.

**Table 1:** Prevalence rates (95% CI) of major birth defects by maternal residence and geographic region: CNPBDSS, 2007–2021

Birth defects	Maternal residence		Geographic region			Total
	Urban	Rural	Eastern	Central	Western	
NTDs	1.3 (1.1–1.4)	2.7 (2.5–3.0)	1.4 (1.3–1.6)	2.2 (2.0–2.5)	2.6 (2.4–2.9)	1.9 (1.8–2.1)
Anencephalus	0.3 (0.2–0.3)	0.6 (0.5–0.7)	0.3 (0.2–0.3)	0.5 (0.4–0.7)	0.6 (0.5–0.8)	0.4 (0.4–0.5)
Spina bifida	0.8 (0.7–0.9)	1.8 (1.7–2.0)	1.0 (0.8–1.0)	1.5 (1.3–1.7)	1.7 (1.5–1.9)	1.3 (1.2–1.4)
Encephalocele	0.2 (0.1–0.2)	0.3 (0.2–0.4)	0.2 (0.2–0.3)	0.2 (0.1–0.3)	0.3 (0.2–0.4)	0.2 (0.2–0.3)
Hydrocephalus	2.7 (2.5–2.9)	3.2 (3.0–3.4)	3.0 (2.8–3.2)	2.7 (2.4–3.0)	3.1 (2.8–3.4)	2.9 (2.8–3.1)
Anotia/microtia	3.0 (2.8–3.2)	3.0 (2.8–3.2)	3.3 (3.1–3.5)	2.4 (2.1–2.7)	3.1 (2.8–3.4)	3.0 (2.8–3.1)
CP	3.4 (3.1–3.6)	2.7 (2.5–2.9)	3.7 (3.5–3.9)	2.8 (2.5–3.1)	2.2 (1.9–2.4)	3.1 (2.9–3.2)
CL/P	5.9 (5.7–6.2)	9.1 (8.7–9.5)	6.9 (6.5–7.2)	7.2 (6.7–7.6)	8.6 (8.1–9.1)	7.4 (7.2–7.6)
CL	2.9 (2.7–3.1)	4.1 (3.8–4.4)	3.4 (3.2–3.6)	3.3 (3.0–3.6)	3.8 (3.5–4.2)	3.5 (3.3–3.6)
CLP	3.0 (2.8–3.2)	5.0 (4.7–5.3)	3.5 (3.3–3.7)	3.9 (3.6–4.2)	4.8 (4.4–5.1)	3.9 (3.8–4.1)
Esophageal atresia/stenosis	0.7 (0.6–0.8)	0.8 (0.6–0.9)	0.8 (0.7–0.9)	0.6 (0.5–0.8)	0.8 (0.6–0.9)	0.7 (0.7–0.8)
Anorectal atresia/stenosis	2.8 (2.6–3.1)	2.3 (2.1–2.5)	3.0 (2.8–3.3)	2.2 (1.9–2.4)	2.2 (1.9–2.4)	2.6 (2.4–2.7)
Hypospadias	5.5 (5.2–5.8)	3.3 (3.1–3.5)	5.9 (5.6–6.3)	3.0 (2.7–3.3)	3.3 (3.0–3.6)	4.5 (4.3–4.6)
Clubfoot	4.6 (4.4–4.9)	4.2 (4.0–4.5)	4.6 (4.3–4.9)	4.3 (3.9–4.6)	4.3 (4.0–4.7)	4.4 (4.3–4.6)
Polydactyly	19.6 (19.1–20.1)	18.3 (17.8–18.9)	20.0 (19.4–20.5)	16.1 (15.4–16.8)	20.4 (19.6–21.1)	19.0 (18.6–19.4)
Syndactyly	6.0 (5.7–6.3)	4.2 (4.0–4.5)	6.2 (5.9–6.6)	4.2 (3.9–4.6)	4.2 (3.8–4.5)	5.2 (5.0–5.4)
LRD	2.2 (2.1–2.4)	2.5 (2.3–2.7)	2.4 (2.2–2.6)	2.2 (2.0–2.5)	2.4 (2.2–2.7)	2.3 (2.2–2.5)
Diaphragmatic hernia	0.6 (0.5–0.7)	0.5 (0.4–0.6)	0.7 (0.6–0.9)	0.4 (0.3–0.5)	0.4 (0.3–0.5)	0.6 (0.5–0.6)
Omphalocele	1.0 (0.9–1.1)	1.0 (0.8–1.1)	1.3 (1.2–1.5)	0.7 (0.6–0.9)	0.6 (0.5–0.7)	1.0 (0.9–1.1)
Gastroschisis	0.3 (0.2–0.4)	0.7 (0.6–0.8)	0.4 (0.3–0.5)	0.5 (0.4–0.7)	0.7 (0.6–0.9)	0.5 (0.4–0.6)
Down syndrome	1.6 (1.4–1.7)	1.2 (1.1–1.3)	1.7 (1.5–1.9)	1.1 (1.0–1.3)	1.1 (0.9–1.3)	1.4 (1.3–1.5)
Total CHDs^a^	55.0 (54.2–55.9)	34.3 (33.5–35.0)	59.3 (58.3–60.3)	27.4 (26.5–28.3)	38.7 (37.6–39.8)	45.4 (44.8–46.0)
VSD	17.7 (17.2–18.2)	10.3 (9.9–10.7)	20.4 (19.8–21.0)	8.9 (8.4–9.4)	8.6 (8.1–9.1)	14.3 (13.9–14.6)
TGA	0.6 (0.5–0.7)	0.5 (0.5–0.6)	0.8 (0.7–0.9)	0.4 (0.3–0.5)	0.3 (0.2–0.4)	0.6 (0.5–0.6)
TOF	0.8 (0.7–1.0)	1.0 (0.8–1.1)	1.2 (1.0–1.3)	0.8 (0.7–1.0)	0.5 (0.4–0.7)	0.9 (0.8–1.0)
ASD/PFO^b^	64.9 (64.0–65.9)	39.2 (38.4–40.0)	69.7 (68.7–70.8)	26.0 (25.2–26.9)	50.5 (49.3–51.7)	53.0 (52.3–53.6)
PDA^c^	29.2 (28.6–29.9)	17.2 (16.7–17.8)	30.9 (30.2–31.6)	12.3 (11.7–12.9)	22.3 (21.5–23.1)	23.6 (23.2–24.1)

Per 10,000 perinatal births

Excluding anotia/microtia, esophageal atresia/stenosis, clubfoot, LRD, diaphragmatic hernia, omphalocele, TGA, and TOF, the prevalence of other birth defects showed statistically significant differences (*P* < 0.05) between urban and rural areas. With the exclusion of encephalocele, hydrocephalus, esophageal atresia/stenosis, clubfoot, and LRD, a statistically significant geographic variation (*P* < 0.05) in the prevalence of the remaining congenital anomalies was observed

*CNPBDSS* China National Population-based Birth Defects Surveillance System, *CI* confidence interval, *NTDs* neural tube defects, *CP* cleft palate, *CL/P* cleft lip with or without palate, *CL* cleft lip without palate, *CLP* cleft lip with palate, *LRD* limb reduction defects, *CHDs* congenital heart diseases, *VSD* ventricular septal defect, *TGA* transposition of great arteries, *TOF* tetralogy of Fallot, *ASD* atrial septal defect, *PFO* patent foramen ovale, *PDA* patent ductus arteriosus

^a^The prevalence of CHDs, excluding cases of isolated ASD/PFO and isolated PDA

^b^ASD and PFO are combined due to the surveillance system's inability to distinguish between them (both coded as Q21.1). PFO: Exclude cases of isolated PFO in preterm infants and cases of isolated PFO in term infants detected after 24 h of birth with a diameter less than 3 mm

^c^The exclusion criteria for PDA are the same as PFO

**Table 2:** Prevalence rates (95%CI) of major birth defects by maternal age and infant sex: CNPBDSS, 2007–2021

Birth defects	Maternal age (y)					Infant sex		Total
	< 20	20–24	25–29	30–34	≥ 35	Male	Female	
NTDs	3.4 (2.4–4.8)	2.9 (2.6–3.2)	1.5 (1.3–1.7)	1.5 (1.3–1.7)	2.4 (2.0–2.9)	1.9 (1.7–2.0)	2.0 (1.8–2.2)	1.9 (1.8–2.1)
Anencephalus	1.1 (0.6–2.0)	0.7 (0.6–0.9)	0.3 (0.2–0.4)	0.3 (0.2–0.4)	0.5 (0.4–0.8)	0.4 (0.3–0.4)	0.5 (0.4–0.6)	0.4 (0.4–0.5)
Spina bifida	2.2 (1.4–3.3)	1.8 (1.6–2.1)	1.1 (0.9–1.2)	1.0 (0.9–1.2)	1.5 (1.2–1.9)	1.3 (1.1–1.4)	1.3 (1.1–1.4)	1.3 (1.2–1.4)
Encephalocele	0.1 (0.0–0.6)	0.3 (0.2–0.5)	0.2 (0.1–0.2)	0.2 (0.2–0.3)	0.3 (0.2–0.5)	0.2 (0.2–0.3)	0.2 (0.2–0.3)	0.2 (0.2–0.3)
Hydrocephalus	4.4 (3.1–5.9)	3.2 (2.9–3.6)	2.6 (2.4–2.9)	3.0 (2.7–3.3)	3.2 (2.7–3.7)	3.1 (2.9–3.3)	2.8 (2.6–3.0)	2.9 (2.8–3.1)
Anotia/microtia	2.9 (1.9–4.2)	2.5 (2.2–2.8)	3.0 (2.7–3.2)	3.1 (2.8–3.4)	3.9 (3.4–4.5)	3.5 (3.2–3.7)	2.5 (2.3–2.7)	3.0 (2.8–3.1)
CP	3.0 (2.0–4.3)	2.9 (2.6–3.3)	3.0 (2.7–3.2)	3.2 (2.9–3.5)	3.4 (2.9–3.9)	2.3 (2.2–2.5)	3.9 (3.6–4.1)	3.1 (2.9–3.2)
CL/P	15.1 (12.7–17.7)	9.4 (8.9–10.0)	6.3 (6.0–6.7)	6.2 (5.8–6.7)	8.5 (7.8–9.4)	8.3 (8.0–8.6)	6.3 (6.0–6.7)	7.4 (7.2–7.6)
CL	6.3 (4.9–8.2)	4.2 (3.9–4.6)	3.0 (2.7–3.2)	3.1 (2.8–3.4)	4.2 (3.6–4.8)	3.8 (3.6–4.1)	3.0 (2.8–3.3)	3.5 (3.3–3.6)
CLP	8.7 (7.0–10.8)	5.2 (4.8–5.6)	3.4 (3.1–3.6)	3.1 (2.8–3.4)	4.4 (3.8–5.0)	4.5 (4.2–4.7)	3.3 (3.1–3.5)	3.9 (3.8–4.1)
Esophageal atresia/stenosis	0.8 (0.4–1.6)	0.7 (0.5–0.9)	0.7 (0.6–0.8)	0.7 (0.6–0.9)	1.1 (0.9–1.5)	0.7 (0.6–0.8)	0.8 (0.7–0.9)	0.7 (0.7–0.8)
Anorectal atresia/stenosis	3.2 (2.2–4.6)	2.6 (2.3–2.9)	2.3 (2.1–2.5)	2.6 (2.3–2.9)	3.7 (3.2–4.2)	3.1 (2.9–3.4)	1.9 (1.7–2.0)	2.6 (2.4–2.7)
Hypospadias	3.4 (2.4–4.8)	3.9 (3.5–4.3)	4.3 (4.0–4.6)	4.9 (4.5–5.3)	5.6 (5.0–6.3)	8.3 (8.0–8.7)	/	4.5 (4.3–4.6)
Clubfoot	7.1 (5.5–9.0)	5.1 (4.7–5.5)	3.9 (3.7–4.2)	4.2 (3.8–4.6)	5.1 (4.5–5.8)	4.7 (4.4–4.9)	4.2 (3.9–4.4)	4.4 (4.3–4.6)
Polydactyly	27.9 (24.6–31.4)	19.9 (19.0–20.7)	17.9 (17.3–18.5)	18.8 (18.1–19.6)	20.3 (19.1–21.6)	21.9 (21.3–22.5)	15.7 (15.2–16.2)	19.0 (18.6–19.4)
Syndactyly	6.9 (5.3–8.7)	4.7 (4.3–5.1)	4.9 (4.6–5.3)	5.6 (5.2–6.0)	5.8 (5.2–6.5)	5.7 (5.5–6.0)	4.5 (4.3–4.8)	5.2 (5.0–5.4)
LRD	2.6 (1.7–3.8)	2.6 (2.3–2.9)	2.2 (2.0–2.4)	2.3 (2.1–2.6)	2.5 (2.1–3.0)	2.6 (2.4–2.8)	2.1 (1.9–2.2)	2.3 (2.2–2.5)
Diaphragmatic hernia	0.8 (0.4–1.6)	0.4 (0.3–0.6)	0.5 (0.4–0.6)	0.6 (0.4–0.7)	0.8 (0.6–1.0)	0.6 (0.5–0.6)	0.6 (0.5–0.7)	0.6 (0.5–0.6)
Omphalocele	1.4 (0.7–2.3)	1.1 (0.9–1.3)	0.9 (0.8–1.1)	0.9 (0.7–1.0)	1.0 (0.7–1.3)	1.0 (0.9–1.2)	0.8 (0.7–1.0)	1.0 (0.9–1.1)
Gastroschisis	2.5 (1.6–3.7)	1.1 (0.9–1.3)	0.3 (0.2–0.4)	0.2 (0.1–0.3)	0.3 (0.2–0.5)	0.5 (0.4–0.6)	0.5 (0.4–0.6)	0.5 (0.4–0.6)
Down syndrome	0.8 (0.4–1.6)	1.0 (0.8–1.2)	0.9 (0.8–1.0)	1.4 (1.2–1.6)	4.5 (3.9–5.1)	1.5 (1.4–1.6)	1.3 (1.1–1.4)	1.4 (1.3–1.5)
Total CHDs^a^	42.2 (38.2–46.5)	34.7 (33.6–35.8)	42.5 (41.6–43.4)	51.5 (50.3–52.8)	66.0 (63.8–68.2)	45.8 (45.0–46.6)	44.9 (44.1–45.8)	45.4 (44.8–46.0)
VSD	13.4 (11.2–15.9)	10.9 (10.3–11.5)	13.6 (13.1–14.1)	15.7 (15.1–16.5)	20.6 (19.4–21.9)	13.0 (12.6–13.5)	15.6 (15.1–16.1)	14.3 (13.9–14.6)
TGA	0.7 (0.3–1.5)	0.6 (0.5–0.8)	0.5 (0.4–0.6)	0.5 (0.4–0.7)	0.6 (0.4–0.8)	0.7 (0.6–0.8)	0.4 (0.3–0.5)	0.6 (0.5–0.6)
TOF	0.8 (0.4–1.6)	0.9 (0.7–1.1)	0.8 (0.7–0.9)	1.0 (0.8–1.2)	1.2 (0.9–1.5)	1.0 (0.9–1.1)	0.8 (0.7–0.9)	0.9 (0.8–1.0)
ASD/PFO^b^	47.2 (43.0–51.8)	39.0 (37.8–40.1)	50.5 (49.6–51.5)	60.2 (58.9–61.6)	76.6 (74.2–79.0)	53.9 (53.0–54.8)	51.9 (51.0–52.8)	53.0 (52.3–53.6)
PDA^c^	21.0 (18.2–24.1)	16.3 (15.6–17.1)	22.5 (21.8–23.1)	27.6 (26.7–28.5)	35.2 (33.6–36.8)	23.8 (23.2–24.4)	23.5 (22.9–24.1)	23.6 (23.2–24.1)

Per 10,000 perinatal births

Excluding CP, LRD, diaphragmatic hernia, omphalocele, and TGA, the prevalence of other birth defects showed statistically significant differences (*P* < 0.05) across maternal age groups. Apart from NTDs (including anencephaly, spina bifida, and encephalocele), hydrocephalus, esophageal atresia/stenosis, diaphragmatic hernia, gastroschisis, Down syndrome,total CHDs, and PDA, the prevalence of other birth defects demonstrated statistically significant differences (*P* < 0.05) between males and females

*CNPBDSS* China National Population-based Birth Defects Surveillance System, *CI* confidence interval, *NTDs* neural tube defects, *CP* cleft palate, *CL/P* cleft lip with or without palate, *CL* cleft lip without palate, *CLP* cleft lip with palate, *LRD* limb reduction defects, *CHDs* congenital heart diseases, *VSD* ventricular septal defect, *TGA* transposition of great arteries, *TOF* tetralogy of Fallot, *ASD* atrial septal defect, *PFO* patent foramen ovale, *PDA* patent ductus arteriosus

^a^The prevalence of CHDs, excluding cases of isolated ASD/PFO and isolated PDA

^b^ASD and PFO are combined due to the surveillance system's inability to distinguish between them (both coded as Q21.1). PFO: Exclude cases of isolated PFO in preterm infants and cases of isolated PFO in term infants detected after 24 h of birth with a diameter less than 3 mm

^c^The exclusion criteria for PDA are the same as PFO

A U-shaped distribution was observed in maternal age-specific prevalence rates for NTDs and anorectal atresia/stenosis. Increased rates were observed in mothers under 20 years, those aged 20–24 years, and those 35 years or older, contrasting with decreased rates in mothers aged 25–29 years and 30–34 years (Table 2). Hydrocephalus, gastroschisis, CL/P, and polydactyly were significantly more prevalent in the youngest maternal age group, while anotia/microtia, Down syndrome, and the total CHDs were more common in the oldest age group (35 years or older). CP had a higher incidence in females, whereas CL/P, anorectal atresia/stenosis, polydactyly, and syndactyly were more prevalent in males (Table 2).

A significant decline in the prevalence of NTDs, hydrocephalus, CL/P, LRD, omphalocele, and Down syndrome was observed, with reductions ranging from 26.7 to 90.6%. The most substantial decreases were noted in NTDs, CL/P, and omphalocele, with AAPC of −16.1% (95% CI −18.4, −13.7), −9.8% (95% CI −11.2, −8.3), and −11.6% (95% CI −18.1, −4.6), respectively. In contrast, total CHDs showed a significant upward trend, with an AAPC of 15.0% (95% CI 11.4, 18.1).

**Fig. 1:**
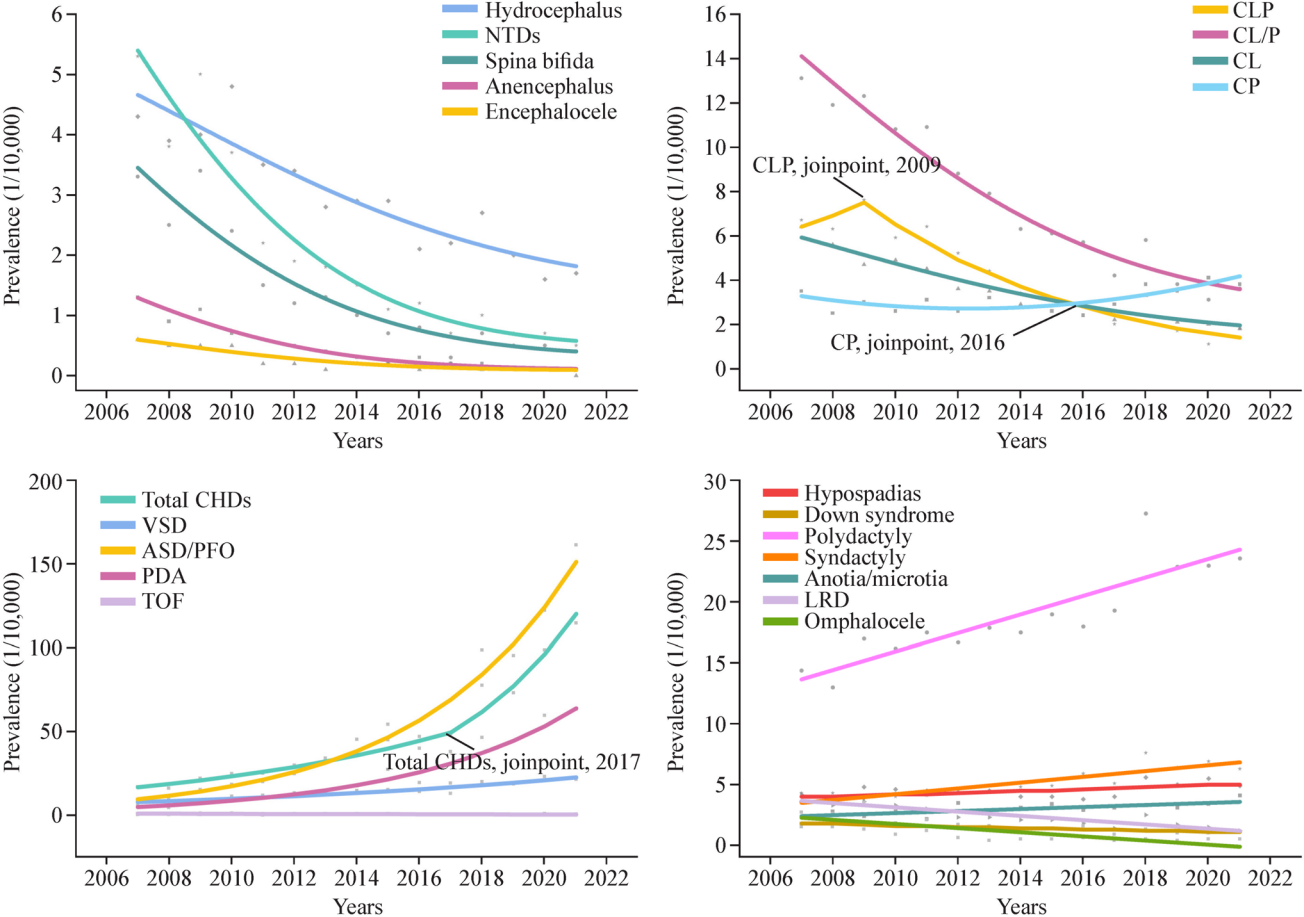
Temporal trends in the prevalence of selected birth defects from 2007 to 2021. ASD and PFO are combined due to the surveillance system's inability to distinguish between them, with both coded as Q21.1. For PFO, cases are excluded if they involve isolated PFO in preterm infants or isolated PFO in term infants detected after 24 h of birth with a diameter less than 3 mm. The exclusion criteria for PDA are the same as those for PFO. Total CHDs represents the prevalence of CHDs excluding isolated cases of ASD/PFO and PDA. Only birth defects showing a significant increasing or decreasing trend are presented here. *ASD* atrial septal defect, *CHDs* congenital heart diseases, *CL* cleft lip without palate, *CLP* cleft lip with palate, *CL/P* cleft lip with or without palate, *CP* cleft palate, *LRD* limb reduction defects, *NTDs* neural tube defects, *PDA* patent ductus arteriosus, *PFO* patent foramen ovale, *TOF* tetralogy of Fallot, *VSD* ventricular septal defect

For CLP, a significant decline from 2009 to 2021 was noted, with an APC of −13.3% (95% CI −15.7, −10.8). Omphalocele exhibited a pronounced decline between 2009 and 2012, with an APC of −44.7% (95% CI −61.7, −20.2). Total CHDs showed a significant upward trend from 2017 to 2021, with an APC of 24.8% (95% CI 16.5, 43.2) (Table 3 and Fig. 1).

**Table 3:** Temporal trends in the prevalence of major birth defects: CNPBDSS, 2007–2021

Birth defects	Crude rate (1/10,000)		AAPC (95% CI)	Trends	
	Year, 2007	Year, 2021		Year	APC (95% CI)
NTDs	5.3	0.5	−16.1 (−18.4, −13.7)		
Anencephalus	1.3	0.1	−18.0 (−20.3, −15.6)		
Spina bifida	3.3	0.4	−15.4 (−18.2, −12.5)		
Encephalocele	0.6	0.0	−14.6 (−18.9, −10.2)		
Hydrocephalus	4.3	1.7	−6.7 (−8.4, −5.0)		
Anotia/microtia	1.8	4.1	2.9 (1.0, 4.8)		
CP	3.5	3.8	2.5 (−0.7, 5.7)	2007−2016	−1.4 (−4.8, 2.2)
				2016−2021	9.8 (1.7−18.6)
CL/P	13.1	3.6	−9.8 (−11.2, −8.3)		
CL	6.4	1.8	−7.9 (−9.4, −6.3)		
CLP	6.7	1.8	−10.4 (−14.8, −5.9)	2007−2009	8.7 (−23.7, 55.0)
				2009−2021	−13.3 (−15.7, −10.8)
Esophageal atresia/stenosis	0.6	0.7	−1.7 (−5.1, 1.9)		
Anorectal atresia/stenosis	2.4	3.6	0.8 (−1.5, 3.3)		
Hypospadias	3.6	4.9	1.7 (0.0, 3.5)		
Clubfoot	2.5	4.5	1.7 (−1.1, 4.5)		
Polydactyly	14.4	23.6	4.1 (2.7, 5.6)		
Syndactyly	4.3	6.3	4.8 (3.1, 6.5)		
LRD	4.2	1.2	−7.1 (−9.1, −5.2)		
Diaphragmatic hernia	0.4	0.5	−2.1 (−4.2, 0.1)		
Omphalocele	2.7	0.5	−11.6 (−18.1, −4.6)	2007−2009	8.8 (−15.0,39.3)
				2009−2012	−44.7 (−61.7, −20.2)
				2012−2021	−1.3 (−5.9, 3.5)
Gastroschisis	1.6	0.2	N/A^a^		
Down syndrome	1.5	1.1	−3.5 (−6.0, −0.9)		
Total CHDs^b^	17.0	114.75	15.0 (11.4−18.1)	2007−2017	11.3 (−5.4, 15.4)
				2017−2021	24.8 (16.5, 43.2)
VSD	5.8	21.6	7.7 (5.6, 9.8)		
TGA	0.4	0.3	0.2 (−8.1, 9.2)		
TOF	0.9	0.7	−4.3 (−6.5, −2.1)		
ASD/PFO^c^	9.5	161.2	21.6 (18.5, 24.8)		
PDA^d^	5.1	63.3	19.7 (15.8, 23.8)		

*CNPBDSS* China National Population-based Birth Defects Surveillance System, *AAPC* average annual percentage change, *CI* confidence interval, *APC* annual percentage change, *NTDs* neural tube defects, *CP* cleft palate, *CL/P* cleft lip with or without palate, *CL* cleft lip without palate, *CLP* cleft lip with palate, *LRD* limb reduction defects, *CHDs* congenital heart diseases, *VSD* ventricular septal defect, *TGA* transposition of great arteries, *TOF* tetralogy of Fallot, *ASD* atrial septal defect, *PFO* patent foramen ovale, *PDA* patent ductus arteriosus

^a^As there were no gastroschisis cases recorded in CNPBDSS in 2019, analysis of this cohort was not applicable

^b^The prevalence of CHDs, excluding cases of isolated ASD/PFO and isolated PDA

^c^ASD and PFO are combined due to the surveillance system’s inability to distinguish between them (both coded as Q21.1). PFO: Exclude cases of isolated PFO in preterm infants and cases of isolated PFO in term infants detected after 24 h of birth with a diameter less than 3 mm

^d^The exclusion criteria for PDA are the same as PFO

## Discussion

This study presents a comprehensive analysis of birth defect prevalence trends in China from 2007 to 2021. By analyzing a nationwide population-based dataset, we identified significant upward or downward trends for various birth defects and revealed epidemiological patterns of major conditions.

Compared with previous data from the Chinese Birth Defects Monitoring Network (CBDMN), the prevalence of selected birth defects in our findings is marginally lower than reported by hospital-based surveillance data [16]. In alignment with the findings of a recent meta-analysis, our study confirms CHDs, polydactyly, and CL/P as leading birth defects. However, we observed significantly higher rates of CHDs and polydactyly, while CL/P prevalence was lower. Notably, NTDs were considerably less frequent in our study (1.9%) compared to Kang et al.’s (12.8%) [17]. Global prevalence rate comparisons are complex due to multiple factors, including differences in ethnicity, socioeconomic status, environmental exposures, and variations in surveillance practices such as diagnostic criteria and study design. In our data, the prevalence of conditions such as anencephaly, LRD, CP, anorectal atresia/stenosis, and hypospadias aligns with that observed in Japan [18]. The prevalence rates of anotia/microtia and CL/P are comparable to those in South Korea [19], but the rates of NTDs, CP, anorectal atresia/stenosis, and CHDs are significantly lower. Conversely, our data show a higher prevalence of anotia/microtia compared to Germany [16], while the rates of NTDs, CL/P, and Down syndrome are substantially lower. Notably, the prevalence of Down syndrome is considerably lower than in the United States and some European countries [3, 18, 20].

Downward trends were observed for NTDs, hydrocephalus, CL/P, LRD, omphalocele, Down syndrome, and TOF during the study period. The decline in central nervous system defects can largely be attributed to national initiatives, such as promoting folic acid supplementation for women of reproductive age [21–23]. In addition, the reduced prevalence of Down syndrome and omphalocele is likely due to the impact of advanced prenatal screening and diagnostic technologies, enabling earlier detection and possible termination of affected pregnancies prior to 28 weeks of gestation [3, 7, 24–27].

In contrast, upward trends were observed for hypospadias, CP, microtia/anotia, polydactyly, syndactyly, VSD, ASD/PFO, and PDA. The increasing prevalence of VSD, ASD/PFO, and PDA likely reflects improvements in diagnostic practices, disease screening, and the routine use of echocardiography, which has facilitated the detection of mild or asymptomatic CHDs during the perinatal period [16, 20]. Additionally, as noted in previous studies, environmental changes may be associated with the incidence of hypospadias [28–30].

Geographical disparities in the prevalence of certain defects are evident, with higher prevalence rates of NTDs and CL/P observed in rural and western regions. These disparities may stem from limited access to folic acid supplementation and inadequate maternal nutrition, especially in rural areas where folic acid-fortified foods and prenatal vitamins are less accessible. Moreover, rural populations may experience greater exposure to pesticides, heavy metals, and pollutants, which could impact the prevalence of these anomalies [31–34]. Discrepancies in prenatal diagnosis rates may also contribute, as urban women generally have better access to prenatal screening and diagnosis, potentially leading to more terminations of pregnancies affected by NTDs or CL/P. The higher prevalence of CP, hypospadias, polydactyly, Down syndrome, and CHDs in urban and eastern regions may be related not only to the extensive implementation of prenatal screening and diagnosis but also to factors such as increasing older maternal age (Supplementary Fig. 1), increased air pollution [35–37], chemical exposure, and distinct climatic conditions [38, 39]. Further research is essential to clarify the intricate relationships and mechanisms driving these regional variations in birth defects prevalence.

Maternal age significantly influences the prevalence of birth defects, with younger mothers more prone to conditions like NTDs, hydrocephalus, CL/P, clubfoot, polydactyly, syndactyly, omphalocele, and gastroschisis [40–42]. Conversely, older mothers exhibit higher rates of CP, hypospadias, Down syndrome, and CHDs. The increased risks associated with teenage pregnancies could be attributed to factors such as underdeveloped growth, physiological immaturity, poorer nutrition, unhealthy lifestyles, lower rates of prenatal screening, and psychosocial stress [43, 44]. For older mothers, the increased likelihood of chromosomal abnormalities and comorbidities, such as diabetes and hypertension, could lead to abnormal fetal cardiovascular development and higher CHD risk [20]. These observations highlight the necessity for age-specific interventions to support maternal health. Additionally, the higher prevalence of microtia/anotia, polydactyly, and syndactyly in male infants is consistent with findings from previous research [8, 16, 45]. The biological and epidemiological mechanisms underlying these trends remain largely unclear and require further research.

This study, while comprehensive, has certain limitations. Firstly, it focuses exclusively on perinatal defects identified at or after 28 weeks of gestation, omitting insights into pregnancy losses or induced abortions before the 28th week. As a result, a definitive understanding of the specific reasons behind observed decreases in incidence rates remains at a speculative level. Secondly, the absence of detailed clinical data precludes a thorough analysis of risk factors such as diabetes, hypertension, smoking, alcohol consumption, and nutritional status. Thirdly, due to the system's coding conventions, certain defects such as ASD and PFO are indistinguishable, precluding more granular analysis and comparison. Despite these limitations, the strength of this study lies in its substantial nationwide population-based sample, which includes over 5 million births and 100,000 infants with birth defects over a 15-year period. This large sample size offers valuable insights into overall trends. Future investigations with more detailed maternal and fetal data could further clarify the underlying mechanisms behind these observed patterns.

In summary, this extensive nationwide population-based study has delineated varied temporal trends in the prevalence of major birth defects in China from 2007 to 2021. A decrease in the prevalence of NTDs, hydrocephalus, CL/P, LRD, omphalocele, Down syndrome, and TOF was observed. In contrast, there was an increase in the prevalence of conditions such as microtia/anotia, hypospadias, polydactyly, syndactyly, and certain types of CHDs. Additionally, the study highlighted significant geographical disparities and maternal age-specific prevalence patterns of certain defects. These findings offer valuable insights into the epidemiological trends and distributions of birth defects, which can inform strategic prevention initiatives and direct future research endeavors aimed at alleviating the impact of birth defects in China.

## Supplementary Information

Below is the link to the electronic supplementary material.

Supplementary file1 (TIF 24893 KB)

## Data Availability

The Chinese Birth Defects Monitoring Network database is not open access publicly available. The corresponding author obtained permission to use the data for this analysis from the National Health Commission of China. The datasets used and analyzed during the study are available from the corresponding author on reasonable request.
