# Supplementary figures and images for: Temporal trends in the prevalence of major birth defects in China: a nationwide population-based study from 2007 to 2021
Source: World J Pediatr. 2024 Nov 2;20(11):1145–54. doi: 10.1007/s12519-024-00844-9 (PMC11582329; doi:10.1007/s12519-024-00844-9)

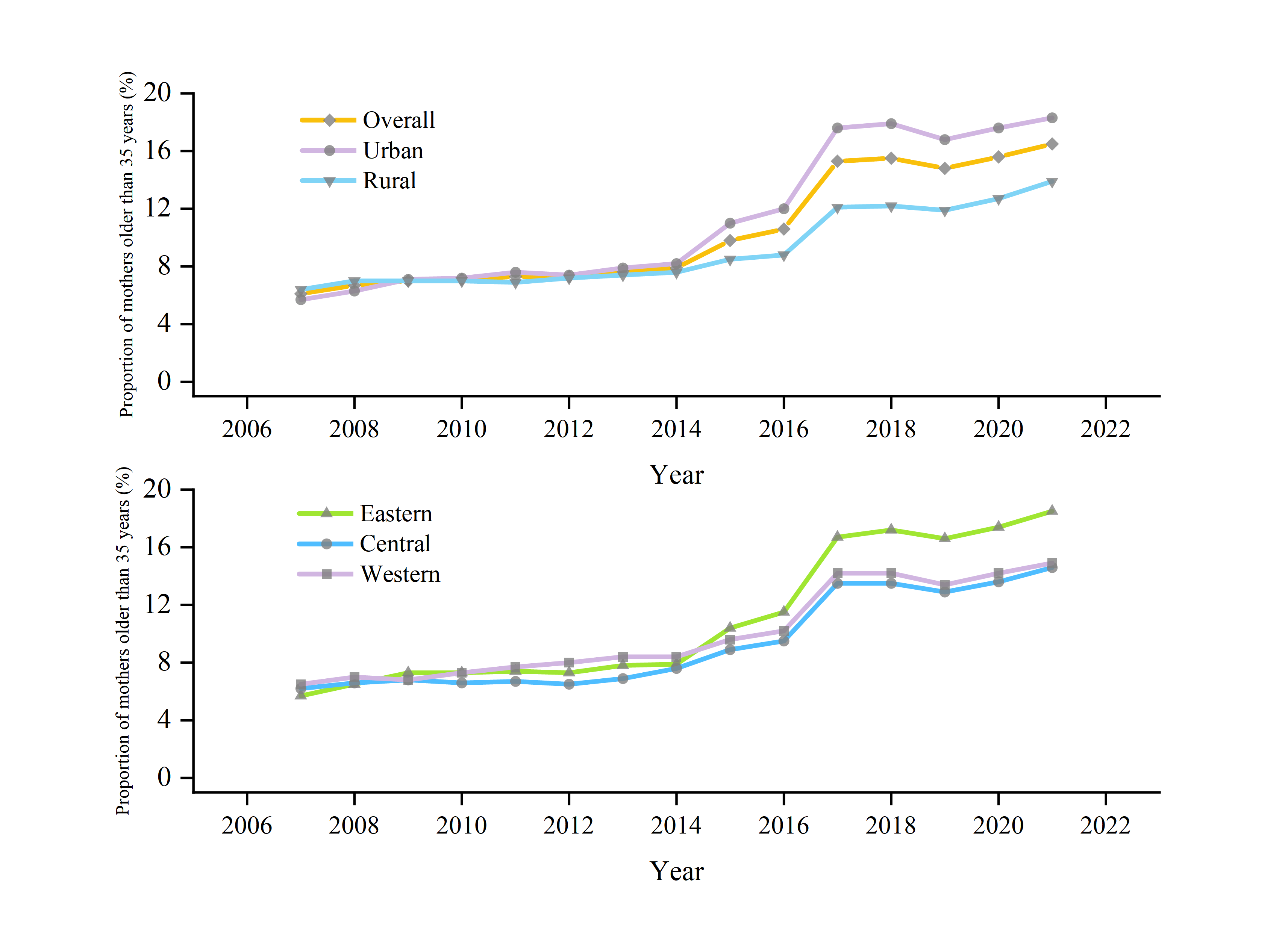

Supplement: Supplementary file 1 — Supplementary file1 (TIF 24893 KB) [file 12519_2024_844_MOESM1_ESM.tif]
